# Analysis of nursing staff job satisfaction and its influencing factors: a cross-sectional study of 38 hospitals/nursing homes in China

**DOI:** 10.3389/fpubh.2025.1526324

**Published:** 2025-03-04

**Authors:** Yu Zhao, Hui-Qun Lu, Yun Xu, Jie-Yu Lu

**Affiliations:** Nantong First People's Hospital, Nantong, Jiangsu, China

**Keywords:** nursing staff, job satisfaction, influencing factors, hospital, nursing homes

## Abstract

**Background:**

Nurses’ job satisfaction directly impacts their attitudes and efficiency at work. This study aimed to identify the factors influencing job satisfaction among nursing professionals in China.

**Methods:**

This cross-sectional study utilized an online questionnaire to assess the job satisfaction, work conditions, and sociodemographic characteristics of nursing professionals in China. Data were analyzed using descriptive statistics, chi-square tests, Spearman correlation analysis, and linear regression analysis.

**Results:**

The study collected 605 questionnaires from 38 hospitals and nursing homes in Nantong. Among them, 599 were valid responses, resulting in a validity rate of 99%. The majority of participants were aged 51–60 (43.14%) and were female (91.65%). The multivariate model results indicated that age, work location, and policy understanding significantly influenced overall job satisfaction. Specifically, the 51–60 age group and those aged ≥61 had a positive impact on overall satisfaction compared to the ≤30 age group (*β* = 0.95, 95% CI = 0.43 to 1.47; *β* = 1.53, 95% CI = 0.82 to 2.25). Compared to working in a hospital, working in a nursing home had a negative impact (*β* = −1.13, 95%CI = −2.10 to −0.17). Additionally, lower policy understanding negatively affected overall job satisfaction.

**Conclusion:**

This study found that age, workplace, and policy understanding are factors influencing the job satisfaction of nursing staff. Therefore, corresponding measures should be taken for young nurses and those working in nursing homes, such as improving welfare benefits and reducing work pressure, to enhance their job satisfaction. In addition, training for all nursing staff should be strengthened to improve their understanding of relevant policies, thereby increasing job satisfaction and retention rates. This will help better meet the growing demand for nursing services.

## Introduction

Nursing is a cornerstone of the healthcare industry, which has long been plagued by a labor shortage (1, 2). The shortage of nursing professionals can be influenced by various factors, with population aging and the increasing burden of chronic diseases being major challenges (3, 4). It is predicted that by 2030, 26.19% of China’s population will be aged 60 or older (5). In India, approximately 21% of the population aged 60 and above suffer from at least one chronic disease, significantly increasing the demand for nursing resources (6, 7). In addition, the World Health Organization predicts that by 2030, there will be a global shortage of 18 million healthcare workers (8). This clearly indicates that it is of great necessity to improve the job satisfaction of healthcare workers and reduce the employee turnover rate.

Job satisfaction is one of the primary concerns for managers and researchers in the nursing environment, as it positively impacts the quality of life at work and the service and care quality of healthcare organizations (9). Job satisfaction measures the extent to which employees are content with their jobs. It is an overall perception of different aspects of work, including tasks, relationships with colleagues, compensation, work environment, and opportunities for growth (9, 10). Employees with high job satisfaction find their work fulfilling, meaningful, and enjoyable, which contributes to improved organizational performance and productivity in the workplace (11). In the healthcare sector, job satisfaction among Canadian nurses has been extensively studied (12, 13). Research has also shown a negative correlation between job satisfaction and nurses’ intention to leave (14). Furthermore, low job satisfaction has been identified as a leading indicator of nurses’ intention to leave in several studies (15, 16). Low job satisfaction can lead to other undesirable outcomes, such as increased absenteeism and burnout among nurses (17), and reduced patient satisfaction with care (18). Therefore, one of the key strategies to overcome future nursing shortages is to retain existing nursing professionals.

Regarding the research on the influencing factors of nurses’ job satisfaction, He et al. found that the monthly salary of community nurses is positively correlated with job satisfaction, while working hours are negatively correlated with satisfaction (19). Wang et al. discovered that age and job position are influencing factors of job satisfaction among nurses in Chinese hospitals (20). A literature review revealed that heavy workload, insufficient resources, poor supervisor relationships, and inadequate training are factors affecting nurses’ job satisfaction (21). Additionally, some research has found that the job satisfaction of nurses in nursing homes is related to the management of supervisors and salary (22). It can be observed that age, salary level, workload, and training frequency are associated with the job satisfaction of nursing staff to some extent. However, there are significant differences in job satisfaction among nursing professionals across different types of healthcare institutions and cultural contexts. For example, private and public hospitals may differ in management styles, compensation, and work environments, which can have varying effects on nurses’ job satisfaction. Additionally, job satisfaction in nursing is influenced by multiple individual and organizational factors. Differences in welfare policies for nursing staff across organizations may also play a role, suggesting that the level of understanding of work-related policies could impact job satisfaction.

In previous studies, most research has focused on hospital nurses, with limited attention given to private nursing homes and similar settings. Moreover, prior research has primarily explored the relationship between individual characteristics, job characteristics, and job satisfaction, while there is a relative lack of studies examining the impact of different work environments and the level of understanding of welfare policies. Therefore, in order to comprehensively understand the influencing factors of job satisfaction among nurses, we distributed a questionnaire to nurses in 38 hospitals and nursing homes in Nantong, China, and constructed a linear model to reveal the main factors affecting job satisfaction. This study provides a scientific basis for the management and policy - making of medical institutions.

## Methods

### Data source

From May to July 2023, a survey was carried out among the nursing staff in 38 hospitals and nursing homes in Nantong, China. It was mainly conducted via the online questionnaire platform “SoJump,” where respondents could submit their answers anonymously. A total of 605 questionnaires were collected. After eliminating 6 invalid ones due to abnormal or blank data, 599 valid questionnaires were obtained, with an effective response rate of 99%.

### Survey method

The questionnaire used in this study was designed independently based on published articles and in consideration of the actual situation of nursing staff in Nantong. Besides basic personal information such as age, gender, and educational level, it also encompasses work - related characteristics, including monthly salary, workplace location, the number of patients served, and working hours. The satisfaction indicators include four satisfaction - related aspects: satisfaction with salary and benefits, satisfaction with the working environment, satisfaction with interpersonal relationships, and satisfaction with professional reputation. The questionnaire adopted a Likert 5-point scale (23), assigning scores of 4, 3, 2, 1, and 0 for very satisfied, fairly satisfied, neutral, fairly dissatisfied, and very dissatisfied, respectively. The overall satisfaction score was calculated by summing the scores from the four satisfaction questions, where a total score of 0–8 indicated dissatisfaction, and 9–16 indicated satisfaction. In addition to the satisfaction questions, the survey also inquired about the respondents’ baseline data and work conditions. The assignment of variables is shown in Table 1.

**Table 1:** Variable assignment table.

Identifier	Variables	Assignment
S1	Satisfaction with salary and benefits	Very dissatisfied = “0”; Dissatisfied = “1”; Neutral = “2”; Satisfied = “3”; Very satisfied = “4”
S2	Satisfaction-working environment	Very dissatisfied = “0”; Dissatisfied = “1”; Neutral = “2”; Satisfied = “3”; Very satisfied = “4”
S3	Satisfaction-interpersonal relationships	Very dissatisfied = “0”; Dissatisfied = “1”; Neutral = “2”; Satisfied = “3”; Very satisfied = “4”
S4	Satisfaction-professional reputation	Very dissatisfied = “0”; Dissatisfied = “1”; Neutral = “2”; Satisfied = “3”; Very satisfied = “4”
S5	Overall satisfaction	Dissatisfied = “1”; Satisfied = “2”
Q1	Age	≤30 = “1”; 31–40 = “2”; 41–50 = “3”; 51–60 = “4”; ≥61 = “5”
Q2	Sex	Male = “1”; Female = “2”
Q3	Education	No = “1”; Elementary school = “2”; Junior high school = “3”; High school = “4”; Undergraduate = “5”; Graduate = “6”
Q4	Place of work	Hospital = “1”; Nursing Home = “2”; Community = “3”
Q5	Hours of work	≤1 = “1”; 2–4 = “2”; 5–7 = “3”; 8–10 = “4”;>10 = “5”
Q6	Working hours	8 h = “1”; 4 h = “2”; 12 h = “3”; 24 = “4”
Q7	Service users	1 = “1”; 2 = “2”; 3 = “3”; 4 = “4”;>5 = “5”
Q8	Salary	1,600–3,000 = “1”; 3,000–5,000 = “2”; 5,000–8,000 = “3”; 8,000–10,000 = “4”; >10,000 = “5”
Q9	Frequency of training	None = “1”; 1/Month = “2”; 1/Quarter = “3”; 1/Self-year = “4”; 1/Year = “5”
Q10	Communicate with clients	Always = “1”; Frequently = “2”; Less = “3”
Q11	Policy understanding	Proficient = “1”; Familiar = “2”; Aware = “3”; Unknowledgeable = “4”

### Statistical analysis

#### Quality control

The reliability of the questionnaire was tested by calculating Cronbach’s alpha. Generally, the Cronbach’s alpha coefficient ranges from 0 to 1. If the reliability coefficient of the scale is above 0.9, it indicates excellent reliability; if the reliability coefficient is between 0.8 and 0.9, it indicates acceptable reliability (24).

The validity of the questionnaire was assessed by calculating the KMO (Kaiser-Meyer-Olkin) measure and performing the Bartlett’s test of sphericity. The KMO statistic is used to check the partial correlations between variables, with values ranging from 0 to 1. The KMO value closer to 1 indicates stronger partial correlations between variables, leading to better factor analysis results. In practice, a KMO value above 0.7 is considered good, while a value below 0.5 is unsuitable for factor analysis (25). The Bartlett’s test of sphericity is used to determine whether the correlation matrix is an identity matrix, i.e., whether the variables have strong correlations. If *p* < 0.05, the test does not follow the spherical distribution, and the hypothesis of variable independence should be rejected, indicating that there are strong correlations between variables. If *p* > 0.05, the test follows the spherical distribution, and the variables are independent, making factor analysis unsuitable (26).

#### Correlation analysis

To explore the preliminary associations between various factors and the job satisfaction of nursing staff, a Spearman correlation analysis was conducted on the four satisfaction measures, overall satisfaction, and other variables in the questionnaire.

#### Factor analysis

In this study, a linear regression model was constructed to analyze potential factors affecting the job satisfaction of nursing staff. First, overall satisfaction was used as the dependent variable, and other variables were used to perform univariate linear regression. Variables with *p* < 0.05 were selected and included in the multivariate linear model. Potential influencing factors were identified through the multivariate linear model. All analyses were implemented using R software (version 4.3.0).

## Results

This study collected 605 questionnaires from 38 hospitals/nursing homes in Nantong, with 599 valid questionnaires, resulting in an effective response rate of 99%. The participants were primarily aged 51–60 years (43.14%) and mostly female (91.65%). The educational level was mainly concentrated at junior high school (37.56%) and senior high school (32.39%); the majority of the participants were from nursing homes (95.72%), and their work experience was primarily in the 2–4 year range (45.08%). Most participants worked 8 hours a day (60.77%). The majority served ≥5 people, had a salary of 3,000–5,000 RMB, and frequently interacted with their clients. Whether in hospitals or nursing homes, most nursing staff receive training once a month and are mostly familiar with relevant policies. Chi-square or Fisher’s exact tests revealed significant differences in satisfaction across different age groups, work locations, daily working hours, number of clients served, training frequency, and policy understanding (*p* < 0.05) (Table 2).

**Table 2:** Descriptive analysis.

Variables		No satisfied	Satisfied	*χ* ^2^	*p*
Age	≤30	61 (38.12)	87 (19.82)	24.27	**<0.001**
	31–40	9 (5.62)	41 (9.34)		
	41–50	22 (13.75)	51 (11.62)		
	51–60	54 (33.75)	206 (46.92)		
	≥61	14 (8.75)	54 (12.30)		
Sex	Male	17 (10.62)	33 (7.52)	1.48	0.224
	Female	143 (89.38)	406 (92.48)		
Education	No	4 (2.50)	7 (1.59)	5.74 ^a^	0.332
	Elementary school	10 (6.25)	48 (10.93)		
	Junior high school	55 (34.38)	170 (38.72)		
	High school	57 (35.62)	137 (31.21)		
	Undergraduate	33 (20.62)	76 (17.31)		
	Graduate Program/Higher	1 (0.62)	1 (0.23)		
Place of work	Hospital	5 (3.12)	20 (4.56)	7.26	**0.027**
	Nursing Home	147 (91.88)	413 (94.08)		
	Community	8 (5.00)	6 (1.37)		
Years of work	≤1	28 (17.50)	68 (15.49)	7.33	0.119
	2–4	78 (48.75)	192 (43.74)		
	5–7	22 (13.75)	105 (23.92)		
	8–10	20 (12.50)	47 (10.71)		
	>10	12 (7.50)	27 (6.15)		
Working hours per day	4 h	96 (60.00)	268 (61.05)	8.64 ^a^	**0.035**
	8 h	3 (1.88)	0 (0.00)		
	12 h	20 (12.50)	49 (11.16)		
	24 h	41 (25.62)	122 (27.79)		
Service users	1	14 (8.75)	26 (5.92)	11.49	**0.022**
	2	5 (3.12)	19 (4.33)		
	3	2 (1.25)	16 (3.64)		
	4	2 (1.25)	30 (6.83)		
	≥5	137 (85.62)	348 (79.27)		
Salary	1,600–3,000	8 (5.00)	24 (5.47)	8.72 ^a^	0.069
	3,000–5,000	126 (78.75)	294 (66.97)		
	5,000–8,000	24 (15.00)	115 (26.20)		
	8,000–10,000	1 (0.62)	3 (0.68)		
	>10,000	1 (0.62)	3 (0.68)		
Frequency of training	None	6 (3.75)	2 (0.46)	11.46 ^a^	**0.022**
	1/Month	115 (71.88) (71.88)	344 (78.36)		
	1/Quarter	31 (19.38)	73 (16.63)		
	1/Self-year	5 (3.12)	9 (2.05)		
	1/Year	3 (1.88)	11 (2.51)		
Communicate with clients	Always	92 (57.50)	292 (66.51)	8.02	**0.018**
	Frequently	60 (37.50)	140 (31.89)		
	Less	8 (5.00)	7 (1.59)		
Policy understanding	Proficient	8 (5.00)	63 (14.35)	28.20	**<0.001**
	Familiar	94 (58.75)	297 (67.65)		
	Aware	50 (31.25)	72 (16.40)		
	Unknowledgeable	8 (5.00)	7 (1.59)		

a Fisher exact test; The value shown in bold is *p* < 0.05.

The reliability and validity of the questionnaire were tested. Reliability was assessed using Cronbach’s alpha, which yielded a value of 0.854, indicating an ideal questionnaire structure with high internal consistency. Validity was assessed using the KMO measure and Bartlett’s test of sphericity. The KMO value was 0.768, and the Bartlett’s test of sphericity yielded a statistic of 1137.73 (*p* < 0.001), indicating that the questionnaire passed the validity test. Therefore, the questionnaire has good reliability and validity (Table 3).

**Table 3:** Questionnaire reliability and validity test.

Test	Test value
Reliability test	
Cronbach’s alpha	0.854
Validity test	
Bartlett test of sphericity	1137.73*
Kaiser-Meyer-Olkin	0.768

*, *p* < 0.001.

**Figure 1:**
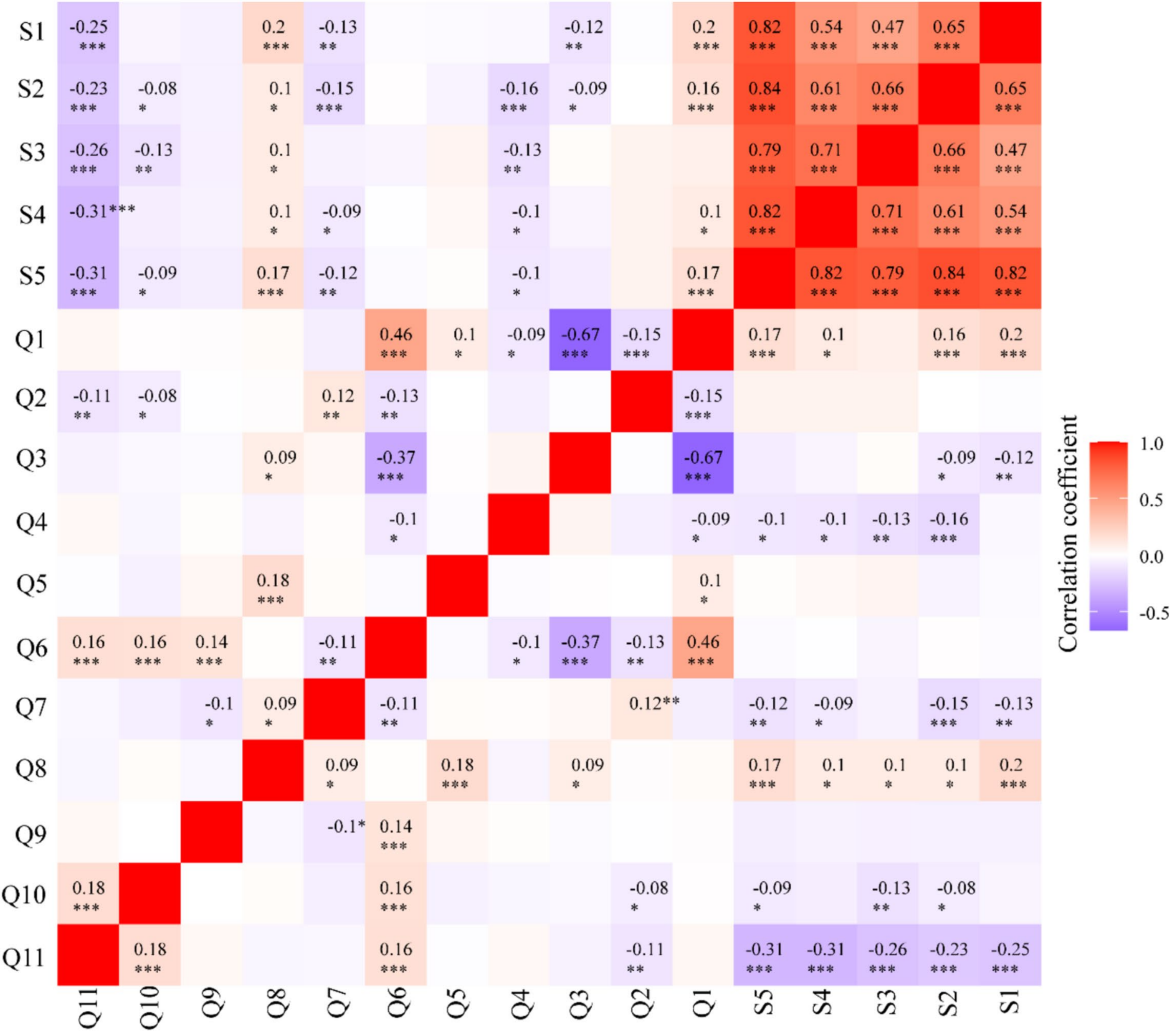
Correlation matrix between satisfaction index and variables. *, *p* < 0.05; **, *p* < 0.01; ***, *p* < 0.001.

To preliminarily explore the associations between satisfaction indicators and various variables, a Spearman correlation analysis was conducted. The results showed that all satisfaction indicators were significantly negatively correlated with policy understanding, meaning that lower policy understanding was associated with lower satisfaction. Additionally, satisfaction indicators were related to salary levels, with higher salaries resulting in higher satisfaction. Overall satisfaction was significantly associated with age, educational level, the number of clients served, and the frequency of interaction with clients. Additionally, age was positively correlated with working hours. Sex was negatively correlated with working hours, the frequency of communication with clients, and the degree of policy understanding. Working hours were positively correlated with the frequency of training, the frequency of communication with clients, and the degree of policy understanding (Figure 1).

Finally, a linear regression model was constructed to further explore the relationships between various influencing factors and overall satisfaction. A univariate linear model was used to screen variables that had a significant impact on overall satisfaction (*p* < 0.05). The univariate model identified Age, Place of work, Working hours per day, Salary, Frequency of training, Communicate with clients, and Policy understanding (Table 4). Subsequently, these variables were included in the multiple linear model, and the model passed the collinearity test (Table 5). The results of the multivariate model showed that age, work location, and policy understanding were significant factors affecting overall satisfaction. Specifically, participants aged 51–60 and ≥ 61 had higher overall satisfaction compared to ≤30 years [*β* = 0.95 (0.43 ~ 1.47); *β* = 1.53 (0.82 ~ 2.25)]. Working in a nursing home had a negative impact on overall satisfaction compared to working in a hospital [*β* = −1.13 (−2.10 ~ −0.17)]. Lastly, lower policy understanding had a negative impact on overall satisfaction (Table 6).

**Table 4:** Single factor regression model.

Variables		SE	*t*	*β* (95% CI)
Age	≤30			(Reference)
	31–40	0.40	2.00	**0.80 (0.02 ~ 1.58)**
	41–50	0.35	1.33	0.47 (−0.22 ~ 1.15)
	51–60	0.25	2.97	**0.75 (0.25 ~ 1.24)**
	≥61	0.36	3.75	**1.34 (0.64 ~ 2.05)**
Sex	Male			(Reference)
	Female	0.37	0.66	0.24 (−0.48 ~ 0.96)
Education	No			(Reference)
	Elementary school	0.81	1.70	1.38 (−0.21 ~ 2.97)
	Junior high school	0.76	1.20	0.92 (−0.58 ~ 2.41)
	High school	0.77	0.85	0.65 (−0.85 ~ 2.15)
	Undergraduate	0.78	0.83	0.64 (−0.89 ~ 2.18)
	Graduate Program/Higher	1.90	0.12	0.23 (−3.49 ~ 3.95)
Place of work	Hospital			(Reference)
	Nursing Home	0.50	−2.28	**−1.15 (−2.14 ~ −0.16)**
	Community	0.82	−2.33	**−1.91 (−3.53 ~ −0.30)**
Years of work	≤1			(Reference)
	2–4	0.29	−1.22	−0.36 (−0.93 ~ 0.22)
	5–7	0.33	0.94	0.31 (−0.34 ~ 0.96)
	8–10	0.39	−1.87	−0.73 (−1.50 ~ 0.03)
	>10	0.47	−0.47	−0.22 (−1.13 ~ 0.70)
Working hours per day	4 h			(Reference)
	8 h	1.43	−1.99	**−2.84 (−5.64 ~ −0.05)**
	12 h	0.32	−2.02	**−0.65 (−1.29 ~ −0.02)**
	24 h	0.23	−0.10	−0.02 (−0.48 ~ 0.43)
Service users	1			(Reference)
	2	0.63	0.45	0.28 (−0.95 ~ 1.52)
	3	0.69	1.81	1.26 (−0.11 ~ 2.62)
	4	0.58	1.48	0.86 (−0.28 ~ 1.99)
	≥5	0.40	−0.97	−0.39 (−1.18 ~ 0.40)
Salary	1,600–3,000			(Reference)
	3,000–5,000	0.45	0.40	0.18 (−0.70 ~ 1.06)
	5,000–8,000	0.48	2.18	**1.05 (0.11 ~ 1.99)**
	8,000–10,000	1.30	0.82	1.06 (−1.48 ~ 3.61)
	>10,000	1.30	1.78	2.31 (−0.23 ~ 4.86)
Frequency of training	None			(Reference)
	1/Month	0.87	2.58	**2.25 (0.54 ~ 3.97)**
	1/Quarter	0.90	1.71	1.54 (−0.23 ~ 3.30)
	1/Self-year	1.09	1.71	1.86 (−0.27 ~ 3.99)
	1/Year	1.09	1.71	1.86 (−0.27 ~ 3.99)
Communicate with clients	Always			(Reference)
	Frequently	0.21	−1.57	−0.34 (−0.76 ~ 0.08)
	Less	0.65	−2.10	**−1.36 (−2.63 ~ −0.09)**
Policy understanding	Proficient			(Reference)
	Familiar	0.30	−5.60	**−1.69 (−2.29 ~ −1.10)**
	Aware	0.35	−7.85	**−2.75 (−3.44 ~ −2.06)**
	Unknowledgeable	0.67	−5.15	**−3.43 (−4.74 ~ −2.13)**

The value shown in bold is *p* < 0.05.

**Table 5:** Multicollinearity test.

Variables	GVIF	Df	GVIF^(1/(2*Df))
Age	1.630	4	1.063
Place of work	2.068	2	1.199
Working hours per day	1.832	3	1.106
Salary	1.451	4	1.048
Frequency of training	1.994	4	1.090
Communicate with clients	1.311	2	1.070
Policy understanding	1.258	3	1.039

**Table 6:** Multiple linear regression model.

Variables		SE	*t*	*β* (95%CI)
Age	≤30			(Reference)
	31–40	0.35	1.95	0.66 (−0.09 ~ 1.41)
	41–50	0.26	3.60	0.67 (−0.01 ~ 1.35)
	51–60	0.36	4.21	**0.95 (0.43 ~ 1.47)**
	≥61	0.38	1.72	**1.53 (0.82 ~ 2.25)**
Place of work	Hospital			Reference
	Nursing Home	0.49	−2.31	**−1.13 (−2.10 ~ −0.17)**
	Community	0.94	−0.55	−0.52 (−2.36 ~ 1.32)
Working hours per day	4 h			Reference
	8 h	1.44	−1.66	−2.40 (−5.22 ~ 0.43)
	12 h	0.32	−1.85	−0.59 (−1.22 ~ 0.04)
	24 h	0.26	−1.07	−0.27 (−0.77 ~ 0.23)
Salary	1,600–3,000			Reference
	3,000–5,000	0.43	−0.33	−0.14 (−0.99 ~ 0.71)
	5,000–8,000	0.47	1.17	0.55 (−0.37 ~ 1.46)
	8,000–10,000	1.25	0.90	1.13 (−1.33 ~ 3.59)
	>10,000	1.31	0.88	1.15 (−1.42 ~ 3.71)
Frequency of training	None			Reference
	1/Month	1.03	1.45	1.50 (−0.52 ~ 3.52)
	1/Quarter	1.04	0.92	0.96 (−1.08 ~ 2.99)
	1/Self-year	1.17	1.03	1.21 (−1.09 ~ 3.50)
	1/Year	1.19	1.25	1.48 (−0.85 ~ 3.80)
Communicate with clients	Always			Reference
	Frequently	0.21	−0.73	−0.15 (−0.56 ~ 0.26)
	Less	0.65	0.09	0.06 (−1.22 ~ 1.34)
Policy understanding	Proficient			Reference
	Familiar	0.31	−4.64	**−1.42 (−2.01 ~ −0.82)**
	Aware	0.36	−6.92	**−2.47 (−3.17 ~ −1.77)**
	Unknowledgeable	0.67	−5.00	**−3.35 (−4.66 ~ −2.04)**

The value shown in bold is *p* < 0.05.

**Figure 2:**
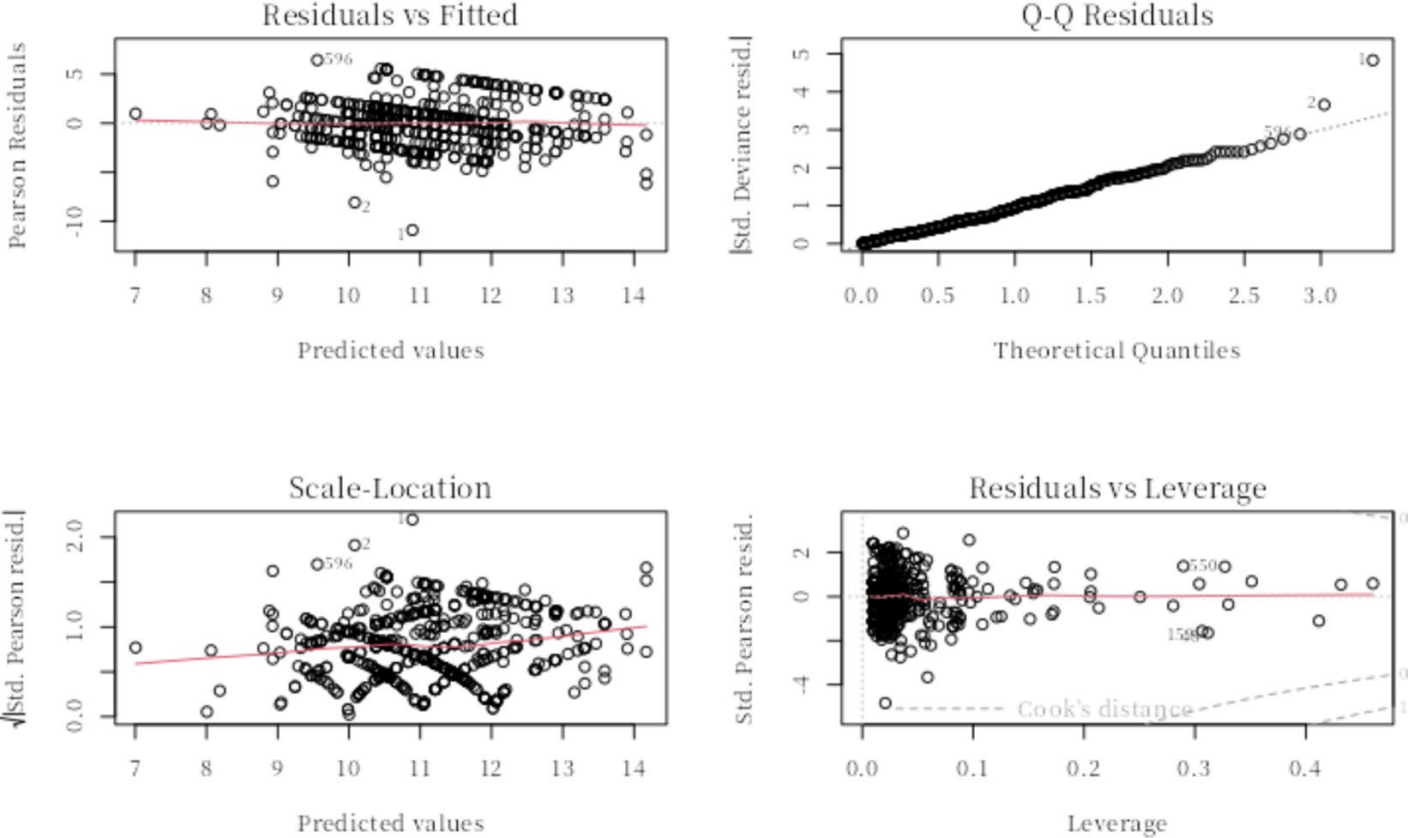
Multiple linear regression model test.

By testing the assumptions of the regression model, it was found that the model could fit the data well. The Q - Q plot demonstrated that the error terms of the model followed a normal distribution. The residual plot indicated that there were no issues with heteroscedasticity in the model (Figure 2).

## Discussion

This study comprehensively investigated the job satisfaction of nursing staff in 38 hospitals and nursing homes in Nantong. The aim was to understand the current situation of nursing staff’s job satisfaction and explore potential influencing factors. A total of 599 valid questionnaires were collected, with an effective response rate of 99%. Such a high effective response rate indicates that the questionnaire was reasonably designed and the survey method was effective, thus laying a solid foundation for the reliability of the research results. The study found that the majority of participants were aged 51–60 years (43.14%) and predominantly female (91.65%), consistent with the findings in Australian studies where women dominate the nursing industry (27). The educational levels were mainly concentrated at the junior high school and senior high school levels (37.56 and 32.39%, respectively). This is similar to the research by GAKI et al. (28). Although no association between educational level and job satisfaction was found in the subsequent analysis of influencing factors, its impact cannot be ignored. A systematic review has found that educational level is related to job satisfaction, but this relationship is inconsistent (29). Among rural healthcare workers, those with a lower educational level tend to have lower job satisfaction (30). This lower educational level may limit their understanding of complex policies and work, thereby affecting their job satisfaction. Since most of the nursing staff in this study were from nursing homes, which have lower recruitment requirements compared with hospitals, the recruitment requirements of nursing homes are lower. Moreover, many nurses in nursing homes have not received nursing - related training, which is similar to the research by Yu et al. The number of nurses in nursing homes accounts for a relatively large proportion of China’s nursing industry (31). To improve job satisfaction, strengthening training and introducing professional talents are urgent issues to be addressed.

Through Spearman correlation analysis, we found that salary levels are positively correlated with job satisfaction. This aligns with the conclusions of numerous domestic and international studies, which suggest that a fair compensation system can significantly enhance job satisfaction and work motivation (32, 33). Moreover, salary levels are closely related to factors such as the work environment and career development, which may also indirectly affect job satisfaction. The study also found that staff who frequently interact with their clients have higher job satisfaction. This is consistent with other research findings, which indicate that good communication not only improves work efficiency but also enhances staff’s sense of identification and value in their jobs (34–36).

To further explore the impact of various factors on overall satisfaction, a linear regression model was constructed. Initially, univariate linear models were used to screen variables that significantly influenced overall satisfaction, and these variables were then included in a multivariate linear model. The results of the multivariate model showed that age, work location, and policy understanding are the primary factors affecting overall satisfaction. Specifically, older staff members reported higher levels of job satisfaction, a finding consistent with previous studies in the healthcare sector (9). This phenomenon can be explained through the lens of Conservation of Resources Theory (37), which suggests that individuals with greater accumulated resources—such as experience, social support, and professional competence-are better equipped to manage workplace stressors and derive greater satisfaction from their roles (38). Older employees may benefit from enhanced job stability, a stronger sense of professional identity, and a deeper understanding of organizational policies, all of which contribute to higher satisfaction levels (39). Furthermore, research by Tourangeau and Cranleyhas shown that older nurses often exhibit greater resilience and adaptability, enabling them to navigate complex work environments more effectively (40). However, it is important to note that this trend contrasts with findings from studies in other industries, where younger employees may report higher satisfaction due to greater enthusiasm for new challenges and opportunities for career growth (41). These divergent findings underscore the importance of contextual factors, such as industry-specific demands and organizational culture, in shaping job satisfaction. By situating our results within this broader theoretical and empirical framework, this study contributes to a more nuanced understanding of the factors influencing job satisfaction among nursing staff.

Staff working in nursing homes reported significantly lower job satisfaction compared to those working in hospitals. This disparity may be attributed to the more complex and challenging work environment in nursing homes, which is often characterized by resource inadequacy and management irregularities (42, 43). These factors can contribute to a decline in job satisfaction, as highlighted by previous studies. The differences in job satisfaction between hospital and nursing home staff reflect broader variations in work scenarios within the healthcare industry and their impact on employee well-being. From a macro perspective, these findings underscore the need to prioritize resource allocation and management improvements in nursing homes, which are often underfunded and understaffed compared to hospitals (44). Enhancing the working environment in nursing homes, optimizing resource distribution, and standardizing management processes are critical not only for improving job satisfaction among nursing staff but also for elevating the quality of elderly care services. This is particularly important in light of the growing demand for elderly care services in aging societies (45). Addressing these issues is essential for promoting the sustainable development of the elderly care sector and ensuring high-quality care for elderly.

Additionally, staff with lower levels of policy understanding exhibited lower overall job satisfaction. This finding aligns with the Job Demands-Resources Model (46), which posits that a lack of resources, including knowledge and clarity about organizational policies, can lead to increased job strain and reduced satisfaction. Insufficient policy understanding may create confusion and uncertainty in daily work, thereby diminishing job satisfaction (47). Therefore, strengthening policy training and improving communication channels are essential strategies for enhancing job satisfaction among nursing staff. Clear and explicit policy guidance enables employees to have clear rules to follow in their work, improving work efficiency and quality (48). At the same time, a robust policy communication mechanism helps to enhance employees’ sense of identity and belonging to the organization, promoting the harmonious and stable development of the healthcare industry (47). By providing higher-quality and more efficient medical services, these measures contribute to the overall improvement of the healthcare system. Therefore, strengthening policy training and communication is of undeniable importance for improving both the service level of the entire medical industry and the job satisfaction of employees.

Although age, work location, and policy understanding were identified as significant influencing factors of job satisfaction in the multivariate analysis, other variables such as salary, training frequency, and working hours also warrant further discussion. While these variables did not show statistical significance in the final model, their potential impact on job satisfaction cannot be overlooked, as they have been widely documented in previous research. Salary is a critical factor influencing job satisfaction, as it directly affects employees’ perceived value and motivation. Although the current study did not find a significant association between salary and job satisfaction, this result contrasts with findings from other studies. For example, a meta-analysis by Judge, demonstrated that compensation is positively correlated with job satisfaction across various industries, including healthcare (49). The lack of significance in our study may be attributed to the relatively homogeneous salary structure among nursing staff in the surveyed institutions, which limits the variability needed to detect significant effects. Future research should explore the role of salary disparities and their impact on job satisfaction in more diverse settings.

Training frequency is another important variable that may influence job satisfaction. Although it was not a significant predictor in our model, previous studies have highlighted the importance of continuous professional development in enhancing job satisfaction among healthcare workers. For instance, Aiken found that nurses who received regular training reported higher job satisfaction due to increased competence and confidence in their roles (50). The absence of a significant relationship in our study may reflect inconsistencies in the quality or relevance of training programs offered to nursing staff. Therefore, improving the quality and accessibility of training programs could be a potential strategy to enhance job satisfaction. Working hours also play a crucial role in shaping job satisfaction, particularly in the healthcare sector, where long and irregular shifts are common. While our analysis did not identify working hours as a significant factor, this finding diverges from studies such as those by Stimpfel, which found that excessive working hours negatively impact nurses’ job satisfaction and overall well-being (51). The discrepancy may be due to differences in sample characteristics or the specific context of nursing homes, where staff may have adapted to longer working hours. Nevertheless, addressing workload and promoting work-life balance remain important considerations for improving job satisfaction in this population. Compared to previous studies, this study integrated nursing staff from communities, hospitals, and nursing homes. In addition to incorporating basic individual and job characteristics, this study also explores the impact of factors such as workplace location and understanding of relevant policies on nursing staff job satisfaction.

This study has several limitations. First, all the hospitals and nursing homes included in the study are located in Nantong, China, and the proportion of nursing staff from nursing homes is relatively high, which may limit the generalizability of the findings. Second, some demographic variables, such as age and years of employment, are likely to be closely related. If one variable is considered in the multivariate analysis, the importance of the other variable may be diminished or obscured. Finally, since the data were collected during a single period, it is not possible to establish a definitive causal relationship.

## Conclusion

Our research findings indicate that age, workplace, and policy understanding are factors influencing the job satisfaction of nursing staff in hospitals and nursing homes in Nantong. Therefore, it is recommended that managers adopt corresponding measures for young nursing staff and those working in nursing homes. For example, improving welfare benefits and reducing work pressure can enhance their job satisfaction. Additionally, training for all nursing staff should be strengthened to improve their understanding of relevant policies, thereby increasing job satisfaction and retention rates. This will contribute to better meeting the ever - growing demand for nursing services.

## Data Availability

The raw data supporting the conclusions of this article will be made available by the authors, without undue reservation.
